# Changes in therapy and survival of metastatic renal cell carcinoma in Estonia

**DOI:** 10.1186/s12885-020-6685-y

**Published:** 2020-03-12

**Authors:** Hannes Jürgens, Kristiina Ojamaa, Helis Pokker, Kaire Innos, Peeter Padrik

**Affiliations:** 1 Tartu University Hospital, Clinic of Hematology & Oncology, Puusepa 8, Tartu, Estonia; 2 University of Tartu, Clinic of Hematology & Oncology, Tartu, Estonia; 3 East Tallinn Central Hospital, Tallinn, Estonia; 4 North Estonian Regional Hospital, Tallinn, Estonia; 5 Department of Epidemiology and Biostatistics, National Institute for Health Development, Tallinn, Estonia

**Keywords:** Metastatic renal cell carcinoma, National cohort, Overall survival, Targeted therapy

## Abstract

**Background:**

Before the era of targeted therapies, cytokines were the main therapy for metastatic renal cell carcinoma (mRCC). Our aim was to analyze the changes in treatments and overall survival (OS) of all mRCC patients in Estonia in relation to the introduction of new medications.

**Methods:**

All patients with mRCC who started medical therapy in Estonia during the years 2004–2012 were identified using the database of the Estonian Health Insurance Fund. Tumor and treatment data were gathered from medical records. Vital status data were obtained from the Estonian Population Registry. The only available therapy before 2008 was interferon alpha-2A (INFa2A), targeted agents added from 2008. For survival analysis, patients were divided into 2 groups: INFa therapy only (group 1) and INFa followed by targeted agents or targeted agents therapy only (group 2).

**Results:**

Out of 416 identified patients, 380 were eligible for analysis. The most common 1st-line treatments were INFa (55%), sunitinib (32%) and INFa+bevacizumab (13%). 28% of patients received 2nd-line therapies and 15% 3rd-line treatments. Median survival of all patients was 13.7 months [95% confidence interval (CI) 11.3–16.2]; 7.6 months (CI 6.4–8.6) for group 1 and 19.8 months (CI 15.6–22.9) for group 2. In multivariate analysis, group 1 had nearly four times higher risk of dying than group 2 [hazard ration (HR) 3.88, 95% CI 2.64–5.72].

**Conclusions:**

The implementation of targeted therapies significantly changed the outcomes of mRCC in Estonia: it prolonged median survival, reduced the risk of death and also enlarged the proportion of patients who received medical therapy.

## Background

Kidney cancer is the fifteenth most common cancer in the world, with more than 400,000 new cases diagnosed in 2018 [1]. Its incidence globally has been rising constantly –from 1990 to 2013 the increase was 2.1 fold [2]. Along with incidence, the mortality of kidney cancer is increasing with estimation of 1,1% more deaths occurring each year [2]. The incidence of kidney cancer in Estonia is among the five highest in Europe and also worldwide [age-standardized (World) incidence in 2018 21.3 per 100,000 in men and 9.7 in women] [1], but survival has been on the average European level (age-standardized relative survival for adult kidney cancers diagnosed in 2000–2007 was 61.1% [95% confidence interval (CI) 57.2–64.6] in Estonia, 60.6 (60.2–61.0) in Europe and 55.8 (55.0–56.6) in Northern Europe) [3]. Data from 2010 to 2014 show further improvement in the 5-year relative survival estimates of kidney cancer up to 65% (62–69) in Estonia [4].

The main therapy of kidney cancer is surgery, but for metastatic disease medical therapy is necessary. The introduction of modern targeted therapies has greatly improved the prognosis of patients with metastatic renal cell carcinoma (mRCC). The multitargeted tyrosine kinase inhibitors (TKIs) sunitinib [5, 6] and sorafenib [7] were the first new therapies approved for advanced RCC and have been available in the European Union since 2006.

In Estonia, interferon alpha-2A (INFa2A) monotherapy, which has been financed by the Estonian Health Insurance Fund, was the standard medical treatment until 2008 [8]. As of 2008, sorafenib was added to the second-line treatment of mRCC. As of the second half of 2009, INFa2A and bevacizumab combination [9] and sunitinib monotherapy are employed as the first-line treatment in patients with low- and intermediate-risk, and temsirolimus [10] in patients with high-risk mRCC. Pazopanib [11] treatment became routinely available from the second part of 2012 and axitinib from 2014 [12].

The effectiveness of the above-mentioned treatments has been evaluated in the randomized prospective trials; nevertheless, there is less information on what kind of effect these treatments have on prolonging survival, taking into account the treatment outcomes of all patients with mRCC.

Danish Renal Cancer Group has evaluated the implementation of targeted therapy in a complete national cohort of patients, showing that this resulted in significantly improved treatment rates and overall survival [13]. Also, Swedish and Norwegian studies reported the contribution of targeted therapies to improved overall survival of mRCC patients [14, 15]. Czech and Dutch studies have analyzed mRCC registry based data on targeted therapies use in their countries [16, 17]. Very recent publication from Sweden suggested that the use of targeted therapies is also increasingly cost-effective over time in mRCC patients [18].

The aim of the current study was to analyze the changes in treatment outcomes of mRCC in Estonia in relation to the introduction of new medications and international comparisons.

## Methods

We used the database of the nation-wide Estonian Health Insurance Fund to identify all patients with mRCC in Estonia who received anticancer treatment with following characteristics: age ≥ 18 years, diagnosis of RCC [malignant neoplasm of the kidney, except renal pelvis, International Classification of Diseases (ICD) version 10 code C64] and anticancer medications prescribed between January 1, 2004 and December 31, 2012. During the study period, all treatments included in this study were reimbursed by the Estonian Health Insurance Fund. The mRCC patients were with metastatic disease diagnosis from 2004 to 2012, including a number of patients with a primary RCC diagnosis before the year 2004. We defined the following cohorts for primary comparison purposes: patients with the diagnosis of mRCC who started treatment from 2004 to 2012 with INFa only (group 1) or INFa followed by targeted agents or targeted agents only (group 2). As new therapies became available from 2008, we set split-point to year 2008 in order to look at two equal time periods (2004–2007 and 2008–2012) too.

For all identified patients we specified their clinical data including treatments and treatment lines from clinical records (both paper and digital records) in three hospitals that provide treatment for mRCC (North-Estonian Regional Hospital, Tartu University Clinic and East-Tallinn Central Hospital). Vital status was updated as of December 31, 2015 from the Estonian Population Registry (3-year follow-up complete), using unique personal identification numbers. The completeness of the study cohort was assessed through comparison with RCC cases registered at the nation-wide population-based Estonian Cancer Registry (2004–2007) that covers the whole country. As new therapies became available from 2008, we analyzed two different treatment periods (2004–2007 and 2008–2012) too. The study protocol was approved by the Ethics Review Committee on Human Research of the University of Tartu and Estonian Data Protection Inspectorate.

The significance of difference between proportions was estimated with chi-squared test. For survival analysis, the patients were followed from the date of treatment initiation until death from any cause or censored at 3 years. Unadjusted Kaplan-Meier survival function was estimated. Median survival (unadjusted) was calculated with 95% confidence intervals and the equality of the medians between variable categories was tested with Pearson chi-squared test. To identify the determinants of survival, univariate and multivariate Cox proportional hazards regression was used to calculate hazard ratios (HR). The statistical significance of the HRs was assessed using 95% confidence intervals. The proportional hazards assumption was tested. All calculations were conducted with STATA 14.1 (StataCorp LP, TX, USA).

## Results

A total of 416 patients were identified, 380 were eligible for analysis. 36 (9.5%) of the identified patients were excluded after the clinical record review due to different reasons (17 patients’ treatment had started treatment before the year 2004; 4 patients had concurrent second metastatic malignancy; 5 had non-RCC histology; 1 was not getting treatment; 9 patients’ medical record data was missing/incomplete). 33% of the patients were identified in group 1 and 67% in group 2.

When comparing the data retrieved from the Estonian Health Insurance Fund with the data from the Estonian Cancer Registry, we did not find any additional mRCC cases in Estonia who received anticancer medical treatment during 2004–2007. However, 43% of RCC with distant metastases cases reported to the Estonian Cancer Registry did not receive medical treatment.

Patient characteristics are provided in Table 1.

**Table 1:** Patient characteristics and median survival from the time of treatment initiation, mRCC patients, Estonia 2004–2012

	Total		Treatment group 1^a^		Treatment group 2^b^		
	N	%	N	%	N	%	*p*-value
Total	380	100	125	100	255	100	
Age group							
15–59	120	32	42	34	78	31	*p* = 0.706
60–69	134	35	45	36	89	35	
70+	126	33	38	30	88	35	
Sex							
Male	252	66	83	66	169	66	*p* = 0.981
Female	128	34	42	34	86	34	
Histology							
Clear Cell	313	82	82	66	231	91	*p* < 0.001
Non-Clear cell	61	16	39	31	22	9	
Unknown	6	2	4	3	2	1	
Nephrectomy							
Done	314	83	102	81	212	83	*p* = 0.712
Not done	61	16	22	18	39	15	
Other	5	1	1	1	4	2	
Treatment started							
2004–2007	137	36	107	86	30	12	p < 0.001
2008–2012	243	64	18	14	225	88	
Prognostic group (only 2008–2012)							
Favorable	50	21	1	6	49	22	*p* = 0.03
Intermediate	153	63	10	55	143	64	
Poor	9	4	1	6	8	4	
Unknown	31	13	6	33	25	11	
Median survival in months (95% CI)	13.7 (11.3–16.2)		7.6 (6.4–8.6)		19.8 (15.6–22.9)		p < 0.001
Survival#							
12-month	53% (48–58)		30% (22–38)		65% (59–71)		
24-month	34% (29–38)		14% (9–21)		43% (37–49)		
36-month	24% (20–28)		9% (5–15)		31% (25–37)		

^a^ INFa only

^b^ INFa followed by targeted agents or targeted agents only

**Fig. 1:**
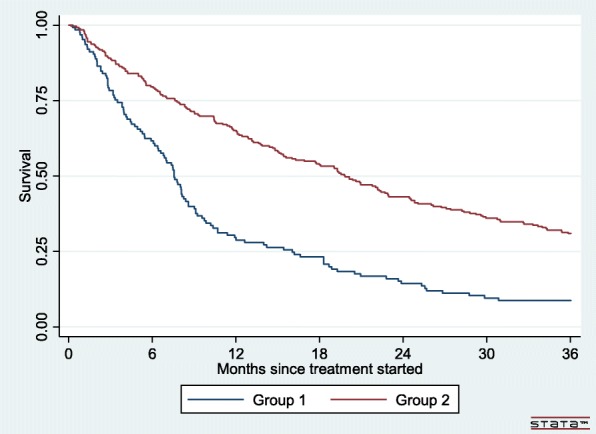
Kaplan-Meier survival curve, mRCC patients, Estonia 2004–2012. Group 1- INFa only. Group 2- INFa followed by targeted agents or targeted agents only

Patients treated with INFa followed by targeted agents or targeted agents only (group 2) had significantly longer median survival compared to patients who received only INFa (group 1) − 19.8 months (CI 15.6–22.9) versus 7.6 months (CI 6.4–8.6), *p* < 0.001(Table 1, Fig. 1).

INFa was the only 1st line treatment in the earlier study period and targeted therapies dominated in the 2nd line of therapy (Table 2). From the year 2008, the usage of targeted therapies increased and by the year 2010 they almost replaced INFa immunotherapy in the 1st-line treatment. Remarkably, more patients received 2nd- and further line treatments in the later treatment period compared to the earlier period (Table 3). The most common 1st-line targeted agent was sunitinib and in the 2nd line sorafenib.

**Table 2:** Overview of 1st- and 2nd-line treatments during the study period, mRCC patients, Estonia 2004–2012

	2004–2007	2008	2009	2010	2011	2012	2008–2012	2004–2012
*First line treatment*	137	26	50	47	58	62	243	380
Immunotherapy INFa (%)	137 (100)	24 (92)	24 (48)	2 (4)	2 (3)	1 (2)	53 (22)	207 (55)
Targeted therapy (%)	0 (0)	2 (8)	26 (52)	45 (96)	56 (97)	61 (98)	190 (78)	190 (50)
INFa+Bevacizumab	0	1	7	17	14	12	51	51 (13)
Sunitinib	0	0	16	26	38	42	122	122 (32)
Sorafenib	0	1	1	1	2	0	5	5 (1)
Temsirolimus	0	0	2	1	2	4	9	9 (2)
Pazopanib	0	0	0	0	0	3	3	3 (1)
*Second line treatment*	4	15	21	23	24	18	101	105
Immunotherapy INFa (%)	0 (0)	0 (0)	0 (0)	0 (0)	0 (0)	1 (6)	1 (1)	1 (1)
Targeted therapy (%)	4 (100)	15 (100)	21 (100)	23 (100)	24 (100)	17 (94)	100 (99)	104 (99)
INFa+Bevacizumab	0	0	0	1	2	4	7	7 (7)
Sunitinib	0	0	2	3	7	1	13	13 (12)
Sorafenib	2	15	18	17	12	12	74	76 (72)
Temsirolimus	0	0	1	2	1	0	4	4 (4)
Pazopanib	2	0	0	0	0	0	0	2 (2)
Everolimus	0	0	0	0	2	0	2	2 (2)

**Table 3:** Distribution of treatment lines over the study years, mRCC patients, Estonia 2004–2012

	2004–2007	2008	2009	2010	2011	2012	2004–2012
*Treatment lines*							
First line	154 (**100)**	26 (**100)**	50 (**100)**	47 (**100)**	58 (**100)**	62 (**100)**	397 (**100)**
Second line	6 (4)	16 (62)	21 (42)	24 (51)	24 (41)	19 (31)	110 (28)
Third line	0 (0)	5 (19)	8 (16)	15 (32)	18 (31)	11 (18)	57 (14)
Fourth line	0 (0)	0 (0)	2 (4)	2 (4)	3 (5)	3 (5)	10 (3)

The median survival (unadjusted) was significantly longer for patients whose tumors were removed (nephrectomy performed) versus not removed and for those who had favorable Memorial Sloan Kettering Cancer Center (MSKCC) prognosis versus intermediate and poor prognosis (Table 4). Non-clear cell histology had a significant negative influence on survival. We observed a statistically not significantly longer survival in the later treatment period versus the earlier, and female versus male. Survival did not differ significantly between age groups (Table 4).

**Table 4:** Median survival from the time of treatment initiation by variable categories, mRCC patients, Estonia 2004–2012

Median survival in months (95% CI)		
Total	13.7 (11.3–16.2)	
Age group		
15–59	11.1 (8.0–17.7)	*p* = 0.667
60–69	14.6 (11.7–20.6)	
70+	13.1 (9.2–19.0)	
Treatment started		
2004–2007	9.7 (8.1–14.8)	*p* = 0.087
2008–2012	15.4 (12.2–19.8)	
Sex		
Male	11.9 (9.1–15.4)	*p* = 0.104
Female	18.3 (12.1–22.9)	
Nephrectomy		
Done	16.0 (13.1–19.5)	p < 0.001
Not done	7.0 (3.8–8.7)	
Histology		
Clear Cell	16.1 (12.9–19.5)	*p* = 0.002
Non-Clear Cell	7.0 (4.7–9.7)	
Prognostic group (only 2008–2012)		
Favorable	28.4 (22.2–55.7)	p < 0.001
Intermediate	13.2 (10.4–16.9)	
Poor	2.4 (1.1–8.5)	
Unknown	15.2 (5.5–21.0)	

Cox proportional hazards regression analysis (Table 5) showed that crude HR for death was 2.29 (95% CI 1.79–2.92) for INFa only group (group 1) compared to INFa plus 2nd-line targeted therapy or targeted therapy only group (group 2) during the 3 year follow-up after treatment started. When adjusted for all variables (including treatment period) in Table 5, the HR for group 1 was even larger (3.88). Interestingly, crude HR for treatment period favored later period but when adjusted for all variables, HR controversially favored earlier treatment period (1.00 vs 2.22).

**Table 5:** Hazard ratios for all-cause mortality during the 3-year follow-up after treatment initiation^a^, mRCC patients, Estonia 2004–2012

	Crude HR	95% CI	Adjusted^b^ HR	95% CI	Adjusted^b^ HR	95% CI
Treatment group						
Group 2^c^	1.00		1.00		1.00	
Group 1^d^	2.29	1.79–2.92	2.15	1.65–2.79	3.88	2.64–5.72
Age group						
15–59	1.00		1.00		1.00	
60–69	0.83	0.63–1.11	0.89	0.67–1.19	0.85	0.64–1.14
70+	0.94	0.71–1.26	0.96	0.71–1.28	0.87	0.64–1.17
Sex						
Male	1.00		1.00		1.00	
Female	0.73	0.57–0.94	0.70	0.54–0.91	0.71	0.55–0.93
Histology						
Clear Cell	1.00		1.00		1.00	
Non clear cell	2.00	1.49–2.69	1.36	0.99–1.87	1.48	1.07–2.04
Nephrectomy						
Done	1.00		1.00		1.00	
Not done	1.90	1.41–2.57	1.62	1.19–2.21	1.53	1.12–2.09
Treatment period						
2004–2007	1.00		–		1.00	
2008–2012	0.81	0.63–1.03	–		2.22	1.52–3.24

^a^ patients with unknown histology (*n* = 6) and other surgery (*n* = 5) excluded

^b^ adjusted for all variables in the table

^c^ treatment with INFa followed by targeted agents or targeted agents only

^d^treatment with INFa only

The risk of dying was higher among patients with non-clear cell histology compared to those with clear cell histology (crude HR 2.00, adjusted HR 1.48), and patients who had not undergone nephrectomy vs those who had (crude HR 1.90, adjusted HR 1.53). Female patients had lower risk of dying than male patients (crude HR 0.73, adjusted HR 0.70).

## Discussion

This study is a comprehensive assessment of mRCC treatments during the era of pretargeted and targeted therapies that used data derived directly from the clinical records of patients. We found that both overall survival (OS) and the hazard ratio (HR) for mortality of the treated patients improved significantly along with the use of new treatments. The only available 1st-line treatment with INFa immunotherapy during the years 2004–2007 was replaced by targeted therapies during 2008–2012. Significantly more patients received 2nd- and 3rd line treatments during the later period.

Until year 2015 results from large randomized trials of various mRCC treatments showed only a significant improvement of progression-free survival (PFS) [5–7, 9, 11, 12] and very seldom improved OS [10] of properly selected mRCC patients. Researchers explained it with cross-over effect and other subsequent effective treatments that confounded the results. Within the past 3 years there is growing evidence that the landscape of treatments is changing. Novel drugs cabozantinib and immonotherapy drug nivolumab were shown to improve OS of mRCC patients after treatment failure with TKI-s [19, 20]. According to more recent publication of phase III trial by Motzer et al. [21] immunotherapy combination of nivolumab and ipilimumab improved the outcome [objective response rate (ORR), OS and PFS] already in first line compared to sunitinib in intermediate and poor prognostic group of mRCC patients. At the time of many proven effective drugs available on the market for mRCC, the question which drug or combination of drugs ultimately give the best result for a specific patient, still remain doctors to decide without clear evidence-based answer.

Among national cohort studies of mRCC, there are 4 trials from Europe: Norwegian [15], Swedish [14] and Czech Republic [16] trial based on data from local registries and a Danish trial that used detailed and complete national data from medical records [13]. Our findings on the improvement of survival and treatment changes are comparable with those trials. For instance, in the Danish report the median OS of treated mRCC patients was 11.5 months in 2008 and 17.2 months in 2010 compared to our median OS of 13.7 months for patients during the whole trial period and 7.6 months for group 1 and 19.8 months for group 2 [13]. There are also similar results on median survival of different risk groups (although the Danish group used Heng and we used MSKCC risk criteria which are somewhat different): 33.4, 18.6 and 5.8 months in the Danish trial compared to our 28.8, 13.2 and 2.4 months for good, intermediate and poor risk patients, accordingly [13]. Nephrectomy was performed in 83% of treated patients (equally in both treatment groups and time periods) which is a much higher rate than previously reported in Scandinavian countries Norway, Sweden and Denmark - 65, 60 and 61%, respectively [13, 14]. Similar nephrectomy rate was reported from the Czech Republic trial (83%) [16]. The patients who had had nephrectomy, had significantly better survival compared with non-operated patients.

Similar findings including improved disease specific survival along with the use of new treatments (targeted therapy era) have been reported from mRCC studies in the US as well [22].

Due to the retrospective design of this trial, there are some limitations. One limitation of this study is the incompleteness of data from medical records (2% of patients were excluded due to major incompleteness of data; 13% of patients had unknown MSKCC risk group status and 2% unknown histology) that might give rise to bias when interpreting results. Yet, survival data are complete due to the reliability and completeness of national registries. There is also a possible selection bias regarding nephrectomy to be more likely performed in patients with better general condition/prognosis and therefore leading to better survival. Selection bias might also be the case when giving 2nd and later line treatment for patients who are in a better condition/prognosis, especially after immunotherapy failure, and poor risk patients who had the only available reimbursed treatment option of INFa until the second half of 2009, while good and intermediate risk patients had 2nd line treatment option with sorafenib from the year 2008. The larger proportion of early period INFa treatment responders proceeding to 2nd line treatment in later period may explain why HR adjusted for all variables, including treatment period, controversially favored the earlier treatment started period to the later. Case numbers in some variable categories were too low to provide reliable results.

Another important fact is that 43% of RCC patients with distant metastases reported in the Estonian Cancer Registry did not receive medical treatment (a finding from data comparison). Unfortunately, there is no data on how many of them were referred for treatment and how many were not referred and what were the reasons for not being referred or not getting treatment. The Danish trial [11] reported that among all referred mRCC patients 29% did not receive treatment due to different reasons (poor PS, patient request, death before the start of treatment, etc.). The proportion of patients not being referred and the reasons thereof remain unknown but we hope that in the future, new effective treatment possibilities will raise the awareness of the broader medical community about the reasonability of medical treatment in mRCC.

Patients who receive targeted therapies have a significant (more than two-fold) prolongation of median survival and reduction in risk of death compared to INFa treatment only, and therefore, this also provides better justification for positive reimbursement decisions in the future for the benefit of patients.

## Conclusions

The results from our Estonian retrospective study of mRCC patients are consistent with findings from the previously reported real-life and national cohort studies from Europe. The implementation of targeted therapies significantly changed the outcomes of mRCC: it prolonged median survival, reduced the risk of death and also enlarged the proportion of patients who received medical therapy.

## Abbrevations

CI: Confidence interval
HR: Hazard ratio
INFa: Interferon alpha
MRCC: Metastatic renal cell carcinoma
ORR: Objective response rate
OS: Overall survival
PFS: Progression-free survival
TKI: Tyrosine kinase inhibitor
US: United States

## References

[1] Ferlay J, Ervik M, Lam F, Colombet M, Mery L, Piñeros M, Znaor A, Soerjomataram I, Bray F (2018). Global Cancer observatory: Cancer today.

[2] Dy GW, Gore JL, Forouzanfar MH, Naghavi M, Fitzmaurice C (2017). Global burden of urologic cancers, 1990-2013. Eur Urol.

[3] Marcos-Gragera R, Mallone S, Kiemeney LA, Vilardell L, Malats N, Allory Y, et al. Urinary tract cancer survival in Europe 1999-2007: Results of the population-based study EUROCARE-5. Eur J Cancer. 2015;51(15):2217-30. 10.1016/j.ejca.2015.07.028 26421824

[4] Innos K, Sepp T, Baburin A, Kotsar A, Lang K, Padrik P, Aareleid T (2019). Increasing kidney cancer incidence and survival in Estonia: role of age and stage. Acta Oncol.

[5] Motzer RJ, Hutson TE, Tomczak P, Michaelson MD, Bukowski RM, Oudard S (2009). Overall survival and updated results for sunitinib compared with interferon alfa in patients with metastatic renal cell carcinoma. J Clinical Oncol.

[6] Motzer RJ, Hutson TE, Cella D, Reeves J, Hawkins R, Guo J (2013). Pazopanib versus sunitinib in metastatic renal-cell carcinoma. N Engl J Med.

[7] Escudier B, Eisen T, Stadler WM, Szczylik C, Oudard S, Siebels M (2007). Sorafenib in advanced clear-cell renal-cell carcinoma. N Engl J Med.

[8] Motzer RJ, Bacik J, Murphy BA, Russo P, Mazumdar M (2002). Interferon-alfa as a comparative treatment for clinical trials of new therapies against advanced renal cell carcinoma. J Clin Oncol.

[9] Escudier B, Bellmunt J, Negrier S, Bajetta E, Melichar B, Bracarda S (2010). Phase III trial of bevacizumab plus interferon alfa-2a in patients with metastatic renal cell carcinoma (AVOREN): final analysis of overall survival. J Clin Oncol.

[10] Hudes G, Carducci M, Tomczak P, Dutcher J, Figlin R, Kapoor A (2007). Temsirolimus, interferon alfa, or both for advanced renal-cell carcinoma. N Engl J Med.

[11] Sternberg CN, Davis ID, Mardiak J, Szczylik C, Lee E, Wagstaff J (2010). Pazopanib in locally advanced or metastatic renal cell carcinoma: results of a randomized phase III trial. J Clin Oncol.

[12] Rini BI, Escudier B, Tomczak P, Kaprin A, Szczylik C, Hutson TE (2011). Comparative effectiveness of axitinib versus sorafenib in advanced renal cell carcinoma (AXIS): a randomised phase 3 trial. Lancet.

[13] Soerensen AV, Donskov F, Hermann GG, Jensen NV, Petersen A, Spliid H (2014). Improved overall survival after implementation of targeted therapy for patients with metastatic renal cell carcinoma: results from the Danish renal Cancer group (DARENCA) study-2. Eur J Cancer.

[14] Wahlgren T, Harmenberg U, Sandström P, Lundstam S, Kowalski J, Jakobsson M (2013). Treatment and overall survival in renal cell carcinoma: a Swedish population-based study (2000-2008). Br J Cancer.

[15] Beisland C, Johannesen TB, Klepp O, Axcrona U, Torgersen KM, Kowalski J (2017). Overall survival in renal cell carcinoma after introduction of targeted therapies: a Norwegian population-based study. Onco Targets Ther.

[16] Poprach A, Bortlíček Z, Büchler T, Melichar B, Lakomý R, Vyzula R (2012). Patients with advanced and metastatic renal cell carcinoma treated with targeted therapy in the Czech Republic: twenty cancer centres, six agents, one database. Med Oncol.

[17] De Groot S, Sleijfer S, Redekop WK, Oosterwijk E, Haanen JB, Kiemeney LA (2016). Variation in use of targeted therapies for metastatic renal cell carcinoma: results from a Dutch population-based registry. BMC Cancer.

[18] Redig J, Dalen J, Harmenberg U, Lindskog M, Ljungberg B, Lundstam S (2019). Real-world cost-effectiveness of targeted therapy in metastatic renal cell carcinoma in Sweden: a population-based retrospective analysis. Cancer Manag Res.

[19] Choueiri TK, Escudier B, Powles T, Tannir NM, Mainwaring PN, Rini BI (2016). Cabozantinib versus everolimus in advanced renal cell carcinoma (METEOR): final results from a randomised, open-label, phase 3 trial. Lancet Oncol.

[20] Motzer RJ, Escudier B, McDermott DF, George S, Hammers HJ, Srinivas S (2015). Nivolumab versus Everolimus in advanced renal-cell carcinoma. N Engl J Med.

[21] Motzer RJ, Tannir NM, McDermott DF, Aren Frontera O, Melichar B, Choueiri TK (2018). Nivolumab plus Ipilimumab versus Sunitinib in advanced renal-cell carcinoma. N Engl J Med.

[22] Pal SK, Nelson RA, Vogelzang N. Disease-Specific Survival in De Novo Metastatic Renal Cell Carcinoma in the Cytokine and Targeted Therapy Era. PLoS ONE. 2013;8(5):e63341. 10.1371/journal.pone.0063341. 10.1371/journal.pone.0063341 PMC3643924 23658823

